# Supplementary material for: Precipitation Dominates the Allocation Strategy of Above- and Belowground Biomass in Plants on Macro Scales
Source: Plants (Basel). 2023 Aug 1;12(15):2843. doi: 10.3390/plants12152843 (PMC10421374; doi:10.3390/plants12152843)
Supplement: Supplementary file 1 [file plants-12-02843-s001.zip › Figure S1-S6.pdf]

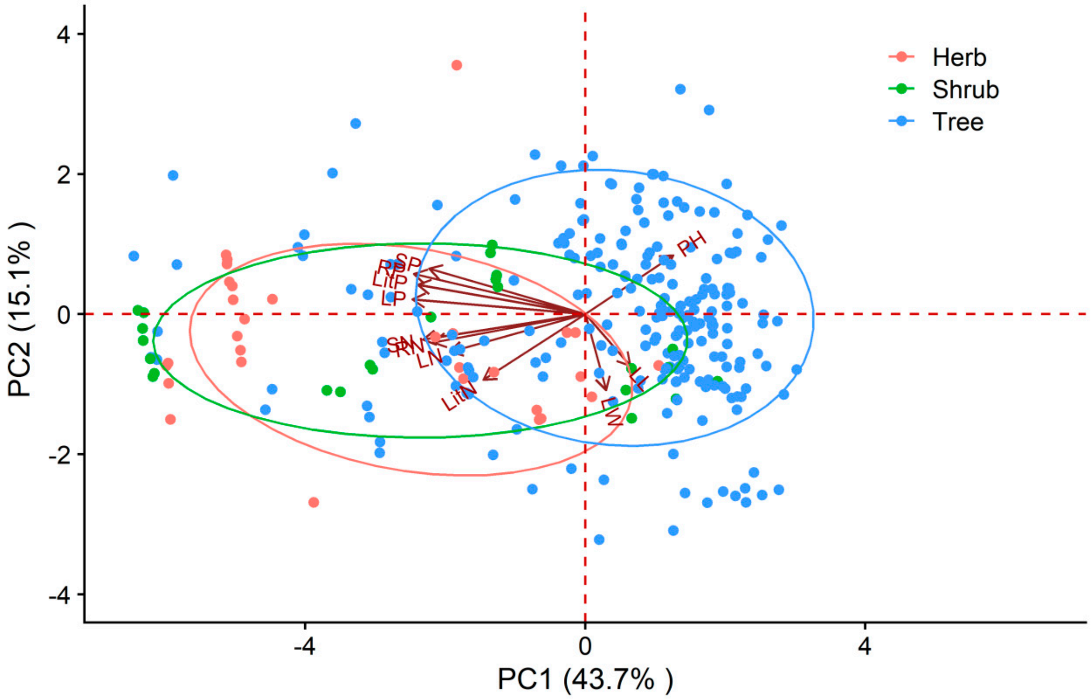

Figure S1. Principal component analysis (PCA) of functional traits of different life types of plants. The data of functional traits include plant height (PH), leaf length (LL), leaf width (LW), leaf nitrogen content (LN), leaf phosphorus content (LP), root nitrogen content (RN), root phosphorus content (RP), stem nitrogen content (SN), stem phosphorus content (SP), litter nitrogen content (LitN), and litter phosphorus content (LitP). Data for all functional traits were log-transformed. The first two principal components explain nearly 48.8% of the variation in functional traits.

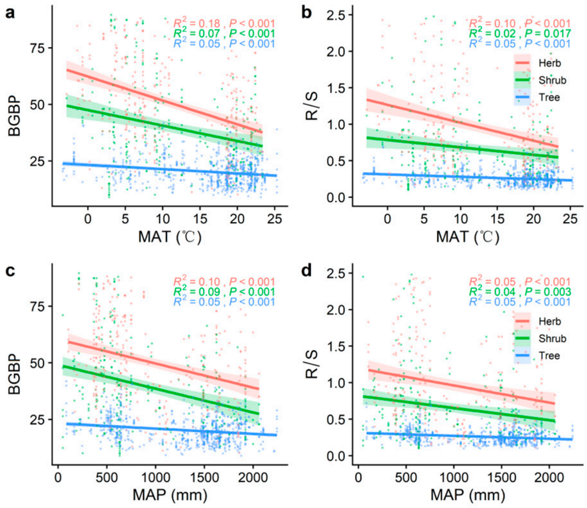

Figure S2. General linear correlation analysis between climate factors and BGBP (%) and R/S of different life types of plants. MAT represents average annual temperature and MAP represents average annual precipitation. R^2 represents goodness of model fitting effect, and *P* value represents significance level.

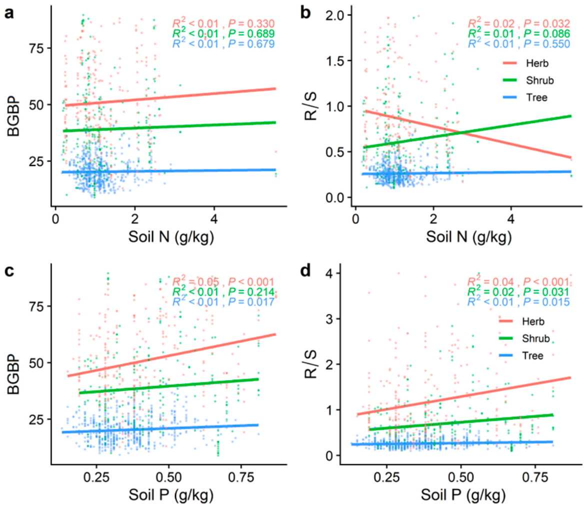

Figure S3. General linear correlation analysis between soil nutrient factors and BGBP (%) and R/S of different life types. Soil N represents Soil nitrogen content and Soil P represents soil phosphorus content. R^2 represents goodness of model fitting effect, and *P* value represents significance level.

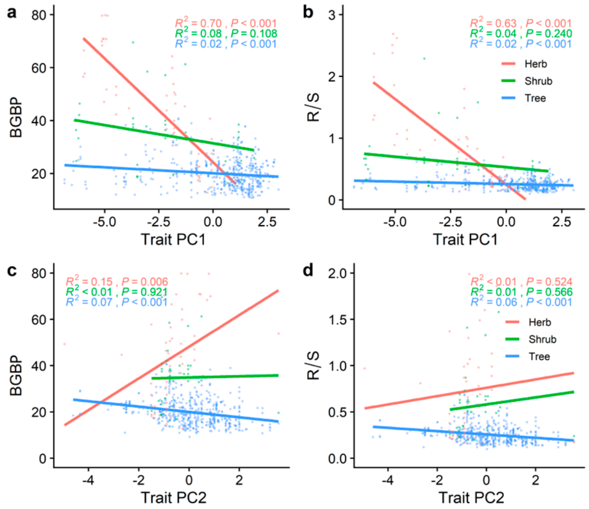

Figure S4. General linear correlation analysis between plant functional traits and BGBP (%) and R/S of different life types. Trait PC1 and Trait PC2 are the first two principal components of functional traits. R^2 represents goodness of model fitting effect, and *P* value represents significance level.

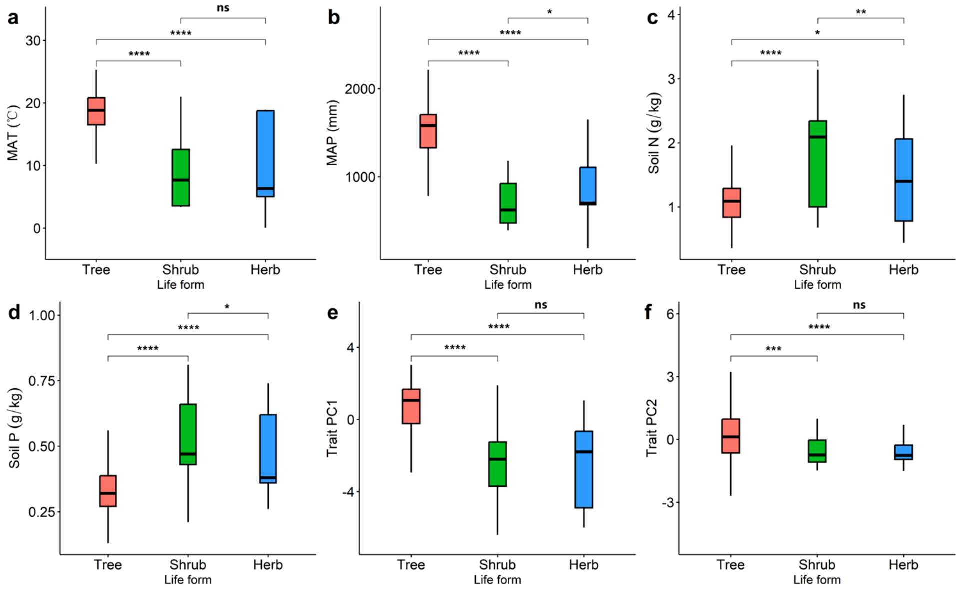

Figure S5. Comparison of the variability of climatic factors (MAT and MAP), soil nutrient factors (soil N content and soil P content), and the first two principal components of functional traits (Trait PC1 and Trait PC2) among trees, shrubs, and herbs plants. Significance levels are denoted as * for *P* < 0.05, *** for *P* < 0.001, **** for *P* < 0.0001, and ns for non-significant differences at the 0.05 level.

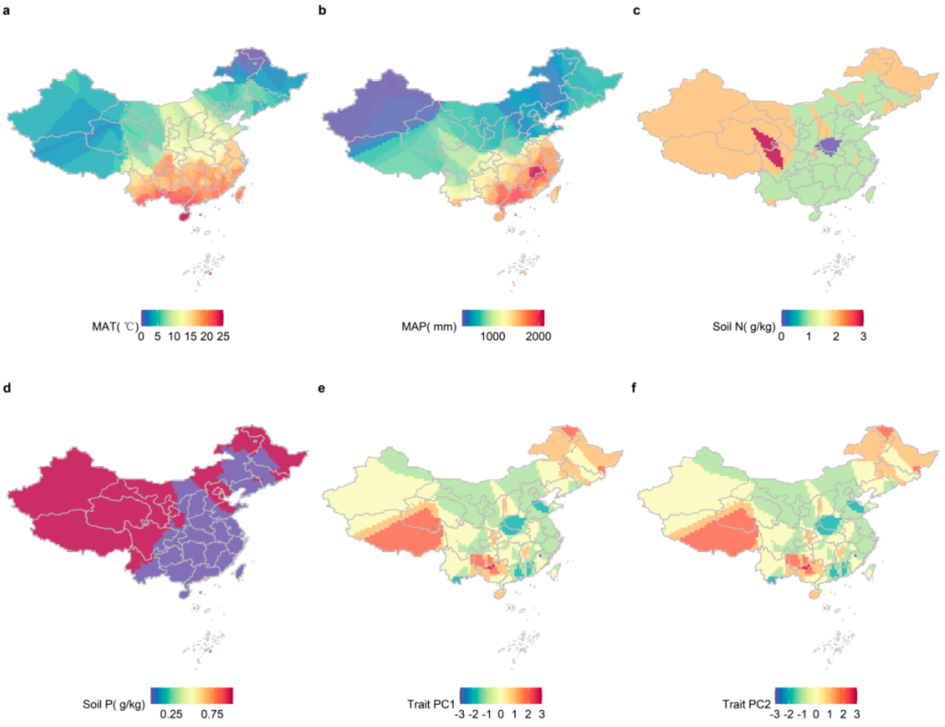

Figure S6. The allocation patterns of each factor in China were studied using kernel density estimation with a spatial resolution of 1×1 km. The factors analyzed included mean annual temperature (MAT), mean annual precipitation (MAP), total soil nitrogen content (Soil N), effective soil phosphorus content (Soil P), and the first two principal components of functional traits (Trait PC1 and Trait PC2).
