# Supplementary material for: Precipitation Dominates the Allocation Strategy of Above- and Belowground Biomass in Plants on Macro Scales
Source: Plants (Basel). 2023 Aug 1;12(15):2843. doi: 10.3390/plants12152843 (PMC10421374; doi:10.3390/plants12152843)
Supplement: Supplementary file 1 [file plants-12-02843-s001.zip › Table S1.pdf]

Table S1 List of cited reference sources

Number	References
1.	Zhong Zebing, Zhou Guoying, Yang Lucun, et al. 2014. The biomass allocation patterns of desert shrub vegetation in the Qaidam Basin, Qing Hai, Chain. Journal of Desert Research**, 34**(4)**,**1042-1048.
2.	Zhou Xiaoguo, Sun Dongjing, Wen Yunguang, et al. 2022. Biomass, productivity and their dynamic changes of different restoration standsin Karst Rocky desertification. Guangxi Sciences**, 29**(1)**,**1-10.
3.	Lu Lihua, Li Hua, Nong You, et al. 2022. Biomass and its distribution pattern in four subtropical plantation ecosystems. Journal of Central South University of Forestry & Technology**, 40**(8)**,**91-98.
4.	Feng Jian, Gao Huilin, Wang Qianchun, et al. 2022. Growth and biomass allocation of Mixed Larch-Ash Forest and its Pure stand in the Eastern Mountainous Area of Liaoning Province. Journal of Northeast Forestry University**, 49**(7)**,**22-27,32.
5.	Guo Yudong. 2021. Study on biomass and carbon density of artificial shrubbery in Kubuqi Desert Area. College of Forestry. Inner Mongolia Agricultural Universit.
6.	Hu Fei-long, Yan Yan, Yu Dandan, et al. 2022. Above and below-ground biomass carbon allocation pattern in Temperate Desert Steppe of Inner Mongolia, China. Journal of Ecology and Rural Environment**, 36**(2)**,**220-226.
7.	Zhang Lei, Lv Guanghui, Jiang Lamei, et al. 2019. Esponse of four species of desert plant biomass distribution to soil factors and allometric growth analysis. Guihaia**, 39**(9)**,**1201-1211.
8.	Zhan Jin, Li Yulin, Han Dan, et al. 2020. Biomass allocation and its ecological significance of three dominant sand-fixing shrubs in the semi-arid desert area. Journal of Desert Research**, 40**(5)**,**149-157.
9.	Lei Longju, Zhao Jinmei, Zhang Xue, et al. 2020. Study on the community characteristics and biomass allocation of different vegetation in the Eastern Qilian Mountains. Pratacultural Science**, 37**(5)**,**853-863.
10.	Meng Yanshan, Meng Lijun, Wang Jingjie, et al. 2019. Models for estimating biomass and its allocation patterns in organs of two major tree species in Qinghai Province. Journal of West China Forestry Science**, 48**(6)**,**21-28.
11.	Cao Zhaoyang. 2021. Biomass distribution pattern and allometric growth model of three common greening tree species in Yangzhou. Academic Research**,** (10)**,**36-38.
12.	Wang Hongbin, Zhang Yukun, Zhang Dacai, et al. 2021. Morphology and biomass allocation of Craex moorcroftii along soil moisture gradient. Acta Agrestia Sinica**, 29**(3)**,**522-530.
13.	Li Zongying, Luo Qinghui, Xu Zhonglin, et al. 2021. Effects of stand density on the biomass allocation and tree height-diameter allometric growth of Picea schrenkiana forest on the northern slope of the western Tianshan Mountains. Arid Zone Research**, 38**(2)**,**545-552.
14.	Zhang Ye, Wei Shuoxing, Jiang Yi, et al. 2020. Structural distribution of

biomass in the 30 years old Keteleeria cyclolepis flous plantation. Chinese Journal of tropical Agriculture, **40**(8),32-40.
15. Gong Ke, Jin Guili, Wang Yuxiang, et al. 2020. Modular biomass of bromus inermis in different areas on the northern slope of Tianshan Mountain. Chinese Journal of Ecology, **39**(8),2629-2635.
16. Chu Bingyin. 2020. Carbon storage characteristics and influencing factors of Chinese fir plantation ecosystem in different age classes. Anhui Agricultural University.
17. Wang Nan. 2020. Effect of slope aspect on biomass and carbon storage of *Vitex negundo* var. heterophylla shrub in low mountains and hilly area of Taihang mountains. HeiBei Agriculture University.
18. Zhang Yaqian. 2020. Community characteristics and biomass distribution of three natural plant species in purple soil of hilly region of central-south Hunan. Central South University of Forestry & Technology.
19. Wang Chenyang. 2020. The calorific value and energy storage of red-heart Chinese Fir plantation in Chenshan forest farm, Jiangxi province. Central South University of Forestry & Technology.
20. Zeng Xaiolin, Wang Dawei, Liu Jingping, et al. 2020. Effects of slope aspect on component traits, biomass structure and water distribution of Arthraxon hispidus. Pratacultural Science, **37**(3),477-485
21. Feng Xing, He Min, Yang Kun, et al. 2020. Study on the reproductive allocation of Arisaema flavum in different altitudes and habitats in the Lhasa Valley. Plateau Science Research, **4**(1),30-36.
22. Qiu Xiaoyu, Xu Zhiyuan, Tu Yangli, et al. 2021. Module biomass and allocation characteristics of invasive plant Tagetes minuta population modules in different habitats. Guihaia, **41**(3),447-455.
23. Du Meiqi, Zhang Hengsuo, Peng Dong, et al. 2020. Distribution of shrub-herb community biomass and its relationship with soil factors in middle and low mountainous areas of northwest Hebei Province. Pratacultural Science, **37**(1),1-9.
24. Lan Jie, Xiao Zhongqi, Li Jiemei, et al. 2020. Biomass allocation and allometric growth of Picea schrenkiana in Tianshan Mountains. Journal of Zhejiang A&F University, **37**(3),416-423.
25. Liu Yuzhen, Cao Wenxia, Wang Jinlan, et al. 2019. Vegetation biomass allocation relationships in the different utilization patterns in alpine meadow. Grassland And Turf, **39**(6),58-65.
26. Guo Lizhu, Huang Ding, Zhang Gong, et al. 2019. Anslysis of biomass allocation and allometric growth of Stellera chamaeiasme in degraded typical steppe. Chinese Journal of Grassland, **41**(6),53-59.
27. Zhang Xuede, Wu Yong, Huang Yuchao, et al. 2019. Root characteristics and biomass allocation of Eucalyptus urophylla × E. grandis plantations of different ages in Leizhou Peninsula. Eucalypt. Science and Technology, **36**(3),22-27.
28. He Xiao, Cao Lei, Xu Shenlin, et al. 2019. Forest biomass characteristics and

influencing factors in different restoration stages in the Daxing'anling forest region of Inner Mongolia, northern China. Journal of Beijing Forestry University, **41**(9),50-58.
29. Cao Meng, Pan Ping, OuYang Xunzhi, et al. 2019. Biomass allocation characteristics and a compatible model for Phoebe bournei in a natural secondary forest. Journal of Zhejiang A&F University, **36**(4),764-773.
30. Li Suhui, Zhao YaJie, Wang Li, et al. 2019. Growth and biomass distribution strategies of two photosynthetic plants in rocky desertification habitats. Journal of Chongqiong Normal University (Natural Science), **36**(4),106-111.
31. Peng Yuhua, Zhang Wei, Shen Wenhui, et al. 2019. Diameter scaling biomass distribution patterns of Castanopsis hystrix plantation in Guangxi. Journal of Hunan Agricultural University, **53**(3),343-348.
32. Ruan Youwei. 2019. Biomass and carbon storage characteristics of overmature pinus massoniana pure forest and its mixed forest in south subtropical zone. Guangxi University.
33. Zhang Shumeng. 2019. Characteristics of the organic carbon and turnover in grassland of different fenced years in southern Ningxia. Northwest A&F University.
34. Wei Xiaocong. 2019. Biomass & nutrient accumulation and distribution of Chenshan Red-heart Chinese-fir, Central South University of Forestry and Technology.
35. Huang Xiuyong. 2017. The biomass structure and nutrient biological cycling of Pinus elliottii plantation on a sandy coastal plain soil. Fujian Agriculture and Forestry University.
36. Zhang Zhen, Jin Guoqing, Zhou Zhichun, et al. 2019. Biomass allocation differences with Pinus massoniana in Guangdong and Hubei provenances. Journal of Zhejiang A&F University, **36**(2),271-278.
37. Hu Xiaoyan. 2019. Carbon storage of Chinese fir plantations at different densities and site indices in Daqing Mountain, Guangxi, Chona. Chinese Academy of Forestry.
38. Shi Fujun, Qing Liqun, Wu Min, et al. 2019. Study on biomass and productivity of medium and large diameter timber of 15-year-old Eucalyptus plantation. Journal of Anhui Agricultural Sciences, **47**(6),113-116.
39. Lin Wanqi, She Hanji, Xue Li, et al. 2019. Allocation patterns of biomass and carbon storage of vegetation layer of a young elaeocarpus sylvestris stand in Xiaoken Forest Farm, Shaoguan City. Ecology and Environmental Sciences, **28**(1),83-89.
40. Ge Lulu, He Zongming, Liu Yu, et al. 2019. Biomass and litter carbon and nitrogen return of different plantation. Journal of Northwest Forestry University, **34**(1),39-46.
41. Luo Shengzhao. 2018. Research on carbon storage and distribution pattern of three artificial forests in Fagaceae. Central South University of Forestry and Technology.

42.	Hou Fang, Wang Keqin, Song Yali, et al. 2018. Carbon storage and distribution in typical forest ecosystems in subalpine of middle Yunnan province. Ecology and Environmental Sciences**, 25**(6),29-35.
43.	Wang Xiaoguang, Wu Yunna, Huo Guangwei, et al. 2018. Effects of grazing on biomass allocations and concentrations of soil nutrients in the grassland of Inner Mongolia. Journal of Desert Research**, 38**(6)**,**1230-1236.
44.	Nie Xiuqing, Xiaog Feng, Li Changbin, et al. 2018. Biomass allocation relationships in the grass layer in alpine shrubland ecosystems on the Tibentan Plateau. Acta Ecologica Sinica**, 38**(18)**,**6664-6669.
45.	Zhang Ji, Zhao Yanli, Wang Yuanzhong, et al. 2018. Biomass allocation and allometry of wild and cultivated Gentiana regescens Franch. Chinese Journal of Ecology**, 37**(12)**,**3584-3589.
46.	Bai Zhiqiang, Li Huan, Wang Wendong, et al. 2018. Biomass model establishment and allocation of dominant tree species in Altai Mountains. Forest Resources Management**,** (4)**,**34-40.
47.	Lu Shiyang, Pan Liping, Peng Wanxia, et al. 2018. Characteristics of biomass allocation in different bamboo plantations. Ecological Science**, 37**(4)**,**123-129.
48.	Meng Peng, Zhang Baixi, Wang Man, et al. 2018. Biomass distribution and architecture of roots in Pinus densiflora and Pinus sylvestris var.mongolica in Horqin sandy land. Chinese Journal of Ecology**, 37**(10)**,**2935-2941.
49.	Yang Zhongyang, Chen Zongzhu, Chen Xiaohua, et al. 2018. Biomass, carbon storage and distribution characteristics of three economic forests in north of Hainan island. Non-wood Forest Research**, 36**(3)**,**62-68.
50.	Zhou Tianyang, Gao Jing, He Jundong, et al. 2018. Plant height, leaf traits, and biomass allocation of three species at heterogeneous slope aspects along a transect in an alpine meadow. Chinese Journal of Applied and Environmental Biology**, 24**(3)**,**0425-0433.
51.	Wang Weifeng，Duan Yuxi, Li Shaobo, et al. 2018. Biomass allocation and soil water content characteristics of three typical shrubs in Mu Us Sandy Land. Journal of West China Forestry Science, 2018**, 47**(03)**,**45-49.
52.	Huang Mingquan. 2018. Comparative analysis and environmental interpretation of the biomass distribution of Simao pine natural forest in typical section. Southwest Forestry University.
53.	Cao Meng. 2018. Tree growth regulation and compatible biomass model for individual tree of natural secondary Phoebe bournei forest. Jiangxi Agricultural University.
54.	She Hanji. 2018. Carbon and nutrient stock in Elaeocarpus sylvestris and Castanopsis fissa ecosystems. South China Agricultural University.
55.	Zheng Xinying. 2018. Study on carbon and nutrient storage in forest ecosystems of Michelia macclurei and Cinnamomum camohora. South China Agricultural University.
56.	Hu Jia. 2018. Research on biomass and carbon storage of dominant plant communities in Poyang Lake wetlands. Journal of Jiangxi Normal University (Natural Science).

57. Wang Yanru. 2018. Research on biomass and soil nutrient content of Cupressus torulosa plantation. Chengdu University of Technology.
58. Tan Guifei, An Jiacheng, Li Guiqing, et al. 2017. Biomass and productivity of 15-year-old Cinnamomum camphora plantation forest. Guangxi Forestory Science**,** **46**(04),369-374.
59. Zhao Panpan, Shao Wenshan, Jin Changqing, et al. 2017. Effects of enclosure on biomass allocation characteristics of Agropyron mongolicum population in desert steppe. Ecology and Environmental Sciences**,** **26**(12)**,**2024-2029.
60. Ming Angang, Liu Shirong, Li Hua, et al. 2017. Effects of close-to-nature transformation on biomass and its allocation in Pinus massoniana and Cunninghamia lanceolata plantations. Acta Ecologica Sinica**,** **37**(23)**,**7833-7842.
61. Wang Fei, Ye Dongmei, Zang Chuanfu, et al. 2017. Tree biomass distribution of Larix gmelinii in Daxing'anling mountains of inner Mongolia. Journal of Northwest Forestry University**,** **32**(5)**,**23-28.
62. Wang Lihua, Gao Jing, Wang Jinniu, et al. 2017. Adaptation of traits and biomass allocation of Pedicularis longiflora to different slope aspects in an alpine meadow. Chinese Journal of Applied and Environmental Biology**,** **23**(4)**,**0648-0657.
63. Chen Rong. 2017. Distribution law of biomass of Magnolia cylindrica at different growth vigor. Protection Forest Science and Technology**,** (6)**,**38-40.
64. Yang Shuo. 2017. Analysis on the growth of Cunninghamia lanceolata, Phoebe and Schima superba mixed forest at different slope positions. Forestry Survey and Design**,** **37**(2),94-97.
65. Sun Yue, He Huaijiang, Li Liang, et al. 2017. Biomass allocation and biomass allometric models of six early-summer herbs under the canopy of broad-leaved Korean pine forest during different growth periods in Jiaohe, Jilin province. Acta Ecologica Sinica**,** **37**(19)**,**6523-6533.
66. Zhang Lili, Wang Zhichao, Chen Shaoxiong, et al. 2017. Biomass allocation pattern of Eucaiyptus × Eucalyptus grandis plantation at different ages. Journal of Northwest A&F University**,** **45**(6)**,**61-68.
67. Sun Meimei. 2017. Carbon sequestration rate and productivity of natural secondary Quercus liaotungensis forests in the Loess hilly region. Northwest A&F University.
68. Hou Yannan. 2017. Carbon stock and allocation patterns analysis among Components in 4 ecological forest in sub-tropical area. Central South University of Forestry and Technology.
69. Qiu Dong, Wu Ganglin, Zhou Xiaobin, et al. 2017. Characteristics of modular traits and interrelationships of the ephemeral species Plantago minuta. Pratacultural Science**,** **34**(4),744-752.
70. Zhao Jing, Wang Xiyuan. 2017. Allocation patterns and allometric relationships between above-and below-ground biomass of three life-form herbaceous plants in the Gurbantunggut Desert. Bulletin of Botanical Research**,** **37**(2),304-311.

71. Meng Huanhuan, Zhang Yuanyuan, Zhou Xiaobing, et al. 2022. Biomass allocation patterns of herbaceous plants in the Gurbantunggut Desert. Journal of Desert Research, **42**(1),96-107.
72. Shen Guiyan. 2017. Biomass and spatial distribution characteristics of Larix olgensis at different stand age in mountain of eastern Liaoning province. Protection Forest Science and Technology, (2),14-16.
73. Li Xinliang. 2017. Biomass and element distribution and accumulation pattern dynamic of plantations of plantations of Shangxi province on Loess plateau. Beijing Forestry University.
74. Dang Xiaohong, Gao Yong, Meng Zhongju, et al. 2017. Biomass allocation patterns and estimation model of five desert shrub species in west ordos region. Journal of Desert Research, **37**(1),100-108.
75. Yan Xiaohong, He Chunlan, Zhou Bing, et al. 2017. Biomass distribution and allometric analysis of bidens frondose relative to growth stage. Journal of Ecology and Rural Environment, **33**(2),150-158.
76. Li Liang, Xia Fucai, Sun Yue, et al. 2017. Biomass allocation of eight early-spring herbs in broadleaf-Pinus koraiensis mixed forest. Journal of Beijing Forestry University, **39**(1),34-42.
77. Wang Yang, Xu Wenting, Xiong Gaoming, et al. 2017. Biomass allocation patterns of Loropetalum chinense. Chinese Journal of Plant Ecology, **41**(1),105-114.
78. Cui Guangshuai, Zhang Lin, Shen Wei, et al. 2017. Biomass allocation and carbon density of Sophora moorcroftiana shrublands in the middle reaches of Yarlung Zangbo River, Xizang, China. Chinese Journal of Plant Ecology, **41**(1),53-61.
79. Sun Yue, He Huaijing, Li Liang, et al. 2017. Biomass and its distribution characteristics of Filipendula palmata under the canopy of mixed coniferous and broad leaved forest in Jiaohe, Jilin Province. Chinese Journal of Applied and Environmental Biology, **22**(6),1055-1062.
80. Jin Yanqiang, Li Jing, Liu Yuntong, et al. 2017. Effects of exclosure on species diversity and biomass allocation of understory vegetation of Savanna ecosystem in Yuanjiang dry-hot valley, Yunnan, Southwest China. Chinese Journal of Ecology, **36**(2),343-348.
81. Zhang Yongjuan, Sun Yan, An Shazhou, et al. 2016. Influences of enclosure on the growth of sagebrush Desert-Seriphidium transiliense. Xinjiang Agricultural Science, **53**(11),2119-2126.
82. Xu Youcai. 2016. Study on biomass and its distribution of 9-year-old Masson Pine Castanopsis eyrei mixed forest. Journal of Green Science and Technology, (19),1-4.
83. Zhu Huinan, He Qiangli, Fang Xiaodong, et al. 2016. Biomass distribution law of Fargesia fargesii in Qinling Mountains. Journal of Green Science and Technology, (18),13-15.
84. Zhu Jiang, Han Hairong, Kang Fengfeng, et al. 2016. Biomass allocation patterns and allometric models of Larix principis-rupprechtii in Mt. Taiyue,

Shanxi. Chinese Journal of Ecology, **35**(11),2918-2925.
85. Liu Chunmei, Bi Jun, Li Liandi, et al. 2016. Biomass of kernel-apricot plantation. Chinese Agricultural Science Bulletin, **32**(25),16-21.
86. Nie Xiuqing, Yang Lucun, Li Changbin, et al. 2016. Patterns of biomass partitioning across alpine shrubs in the Three-river Source Region. Chinese Journal of Applied and Environmental Biology, **22**(4),0538-0545.
87. Lie Zhiyang, Zhou Tongtong, Xue Li, et al. 2016. Effect of density on biomass allocation of acacia auriculaeformis stands. Hunan Forestry Science & Technology, 43(4),86-89.
88. Zhang Sen, Xie Zhiyin, Chen Leixi, et al. 2016. Biomass and annual growth of different aged rubber plantations mixed with Rauvolfia vomitoria and Flemingia macrophylla in Xishuangbanna. Chinese Journal of Ecology, **35**(7),1704-1712.
89. Zhang Jian. 2016. Research on species diversity, biomass and carbon storage of different plantations in Guangxi karst area. Guangxi University.
90. Wu Lisu. 2016. Research on biomass, carbon reserve and nutrient elements in eucalyptus plantation under returning farmland to forest. Guangxi University.
91. Gao Jie, Guo Zijian, Liu Yanhong, et al. 2016. Biomass allocation pattern and its influencing factors across natural Chinese pine forests of different ages in Songshan, Beijing. Chinese Journal of Ecology, **35**(6),1475-1480.
92. Song Qinghua. 2016. The environment adaptability of biomass allocation and root functional traits of Stipa krylovii in degraded alpine grassland. Northwest Normal University.
93. Ji Lei. 2016. Biomass and diversity of shrubs and herbs under three types different canopy forests in Jingouling forestry station. Beijing Forestry University.
94. Fu Yao. 2016. Quantitation of biomass and carbon storage for Chinese fir plantation. Beijing Forestry University.
95. Fan Chunnan, Cheng Yan, Zheng Jinping, et al. 2016. On spring ephemeral plants biomass (II)—biomass and distribution characteristics of anemone raddeana. Journal of Beihua University, **17**(1),24-29.
96. Yu Zhining, Guo Zengqiang, Li Liang, et al. 2016. Biomass allocation research of Aegopodium alpestre. Journal of Jilin Forestry Science and Technology, **45**(1),33-35.
97. Li Jing, Wang Linghong, Cheng Dongliang, et al. 2016. Above and below ground biomass allocation on herb layer of natural evergreen broad-leaved forest and Chinese fir plantation with different age classes. Journal of Nanjing Forestry University (Natural Sciences Edition), **40**(5),170-176.
98. Wu Wei. 2016. Study on the biomass and its distribution of Chinese Fir plantations in Fujian province. FuJian Forestry, (5),39-42.
99. Li Zhaofeng, Huang Bihua, Fu Chengjie, et al. 2015. Comparative study on the biomass allocation pattern and productivity of seven different Fokienia hodginsii mixed forest. Journal of Wuyi University, **34**(9),1-5.

100. Wang Wendong, Bai Zhiqian, Alimu Maimaiti, et al. 2016. Biomass comparison and estimation models for six dominant species of woody shrubs in the forest zones of the Tianshan Mountains. Acta Ecologica Science**, 36**(9)**,**2695-2704.
101. Huang Yunfeng, Liu Yi, Huang Shiyou, et al. 2015. A study of effect of different forest ages on growth and biomass allocation of Masson's pines. Journal of Sichuan Forestry Science and Technology**, 36**(4)**,**72-75.
102. Zhang Sheng, Xiang Wenhua, Ou Yangshuai, et al. 2015. Forest biomass and allocation pattern in 4 subtropical forests in Hilly region of central Hunan Province. Guangxi Forestry Science**, 44**(2)**,**104-109.
103. Xiong Jiangbo. 2015. A study on biomass and carbon storage of the five specise plantations in subtropical China. Guangxi University.
104. Ma Xiaobing. 2015. Study on the response of the biomass of Gentianae on the eastern of Qinghai-Tibetan plateau to the altitude and environmental factors. Northwest Normal University.
105. Shen Jiapeng, Zhang Wenhui, Li Yanhua, et al. 2015. Biomass and carbon density and patterns of their distribution in the plantations of robinia pseudoacacia and Pinus tabulaeformis in Loess Gullies in Eastern Gansu. Science Silvae Sinicae**, 51**(4)**,**1-7.
106. Song Bianlan. 2015. Biomass, carbon and nitrogen pool, and carbon sequestration of two typical forest ecosystems in Loess Plateau Hilly Region, China. University of Chinese Academy of Sciences.
107. Li Yunlong, Gong Hede. 2015. Characteristics of community structure and biomass distribution of rhododendron adenogynum shrub in Laojunshan Mountain. Journal of West China Forestry Science**, 44**(10)**,**121-124.
108. An Yu, An Hui. 2015. Response of plant growth and biomass allocation to grazing disturbance in desert steppe of Ningxia. Acta Botanica Boreali-Occidentalia Sinica**, 35**(2)**,**373-378.
109. Ming Angang, Zheng Lu, Ma Jing, et al. 2015. Biomass, carbon stock and allocation characteristics in Mesua ferrea plantation. Journal of Beijing Forestry University**, 37**(2)**,**32-39.
110. Wang Qi, Cao Yiming, Guo Jinping, et al. 2015. Biomass and productivity of six main vegetations in Guandishan region, Shanxi China. Journal of Shanxi Agriculture University**, 35**(1)**,**432-437.
111. Li Liang, Sun Yue, Xia Fucai, et al. 2014. Analysis of biomass allocation of Meehania fargesii. Journal of Beijing University**, 15**(6)**,**804-807.
112. An Hui. 2014. Effect of grazing on morphological plasticity and biomass allocation of dominant species in desert steppe. Journal of Arid Land Resources and Environment**, 28**(11)**,**116-121.
113. Qiu Dong, Zhou Guiling, Liu Tongye. 2014. Analysis of biomass allocation and allometric growth of three Sterigmostemum species in Junggar Basin. Agricultural Research in the Arid Areas**, 32**(6)**,**215-220.
114. Zhang Haiyan, Shen Xiangjin, Li Fei, et al. 2014. Partitioning of organic carbon in Leymus chinensis grossland ecosystems with different population

densities in Songnen grassland, China. Chinese Journal of Ecology, **33**(9),2281-2287.

115. Zheng Lu, Cai Daoxiong, Lu Lihua, et al. 2014. Biomass allocation of different species plantations in subtropical area of China. Forest Research, **27**(4),454-458.

116. Wang Juhong, Chen Wen. 2014. Comparative study of biomass of modules and morphological plasticity of four Asteraceae species at the flowering stage. Chinese Journal of Ecology, **33**(8),2031-2037.

117. Li Gang, Zhao Xiang, Zhang Binbin, et al. 2014. Biomass allocation pattern and estimation model building of Caragana korshinskii with different plant heights. Acta Agrestia Sinica, 22(4),769-775.

118. Bai Zhiqiang, Liu Hua, Fang Yue, et al. 2014. The biomass distribution pattern of coniferous forest in the Kanas Nation Nature Reserve. Journal of Agricultural University of Heibei, **37**(4),14-19.

119. Dong Dian, Lin Tianxi, Tang Jingyi, et al. 2014. Biomass allocation patterns and allometric models of Tilia amurensis. Journal of Beijing Forestry University, **36**(4),54-63.

120. Liu Ming, Tang Cuiping, Guo Feng, et al. 2014. A study on the population dynamics and its biomass adjustment mechanisms ofclonal plant Hippophae rhamnoides L. ssp. sinensis. Journal of Nanjing Forestry University (Natural Sciences Edition), 38(04),57-63.

121. Wu Ming. 2014. Nutrient use efficiency and nutrient return dynamics of Michelia hedyosperma plantation. Guanxi University.

122. Yan Xiaohong, Zhou Bing, Wang Ning, et al. 2014. Module biomass structure traits of the alien invasive plant ageratum conyzoides. Journal of Jinggangshan University (Natural Science), **35**(3),101-106.

123. Hou Zhaojiang. 2014. The spatiotemporal differences of biomass allocation pattern of Melica przewalskyi in degraded grassland. Northwest Normal University.

124. Ding Jianlin, Han Yue, Bao Weikai, et al. 2014. Biomass allocation strategies of iomass allocation strategies of Lilium regale ilium regaleand their altitudinal effects. Chinese Journal of Applied and Environmental Biology, **20**(2),254-260.

125. Tao Ye, Zhang Yuanming. 2014. Biomass allocation patterns and allometric relationships of six ephemeroid species in Jungger Basin, China. Acta Prataculturae Sinica, **23**(2),38-48.

126. Gao Qiao, Yang Xiaocheng, Yin Chunying, et al. 2014. Estimation of biomass allocation and carbon density in alpine dwarf shrubs in Garze Zangzu Autonomous Prefecture of Sichuan Province, China. Chinese Journal of Plant Ecology, **38**(4),355-365.

127. Wang Ning. 2014. Study on distribution patterns of carbon density and carbon stock in the forest ecosystem of Shanxi. Beijing Forestry University.

128. Zhao Jinlong, Wang Lonxin, Han Hairong, et al. 2014. Biomass and spatial distribution characteristics of Pinus tabulaeformis naturalsecondary forest

at different age groups in the Liaoheyuan Nature reserve, Hebei Province. Acta Ecologica Sinica**,** **43**(23)**,**7026-7037.
129. Zhao Lu, Guan Kaiyun, Li Wenjun, et al. 2014. Phenotypic plasticity and biomass allocation of Iris tenuifolia pall, in different habitats. Chinese Journal of Ecology**,** **33**(3)**,**618-623.
130. Pan Peng, Lv Dang, Ouyang Xunzhi, et al. 2014. A study on biomass and carbon storage of natural Pinus massoniana forest at different stand growing stages in central Jiangxi province. Acta Agriculturae Universitatis Jiangxiensis**,** **36**(1)**,**131-136.
131. Li Xuewen. 2013. Study on the biomass, nutrient content and cnreserves of Betula alnoides piantation. Guanxi University.
132. Zhang Hao, Song Tongqing, Wang Kelin, et al. 2013. Oak biomass and its allocation at different stand ages in West of Guangxi, China. Research of Agricultural Modernization**,** **34**(6)**,**758-762.
133. He Hongzao, Song Jiuxiang, Liu Yingying, et al. 2013. Study on the biomass and allocation of Chinese fir forest in Southeast of Guizhou. Guangxi Agricultural Science**,** **40**(21)**,**58-60.
134. Yang Haotian, Li Xinrong, Liu Lichao, et al. 2013. Biomass allocation patterns of four shrubs in desert grassland. Journal of Desert Research**,** **33**(5)**,**1340-1348.
135. Yu Yuefeng, Song Tongqing, Zeng Fuping, et al. 2013. Dynamic changes of biomass and its allocation in Cunninghamia lanceolata plantations of different stand ages. Chinese Journal of Ecology**,** **32**(7)**,**1660-1666.
136. Li Chunyi. 2013. Biomass partitioning of lacebark pine and its tree-ring growth in relation to climatic factors. Beijing Forestry University.
137. Li Bin, Tang Guoyong, Li Kun, et al. 2013. Vegetation biomass allocation and its spatial distribution after 20 years ecological restoration in a dry-hot valley in Yuanmou, Yunnan Province of Southwest China. Chinese Journal of Applied Ecology**,** **24**(6)**,**1479-1486.
138. Wang Juan, Chen Yunming, Cao Yang, et al. 2013. Carbon concentration and carbon storage in different component of natural Quercus wutaishanica forest in Ziwuling of plateu, northwest China. Chinese Journal of Ecology**,** **31**(12)**,**3058-3063.
139. Wang Ning, Wang Baitian, Wang Ruijun, et al. 2013. Biomass allocation patterns and allometric models of Populus davidiana and Pinus tabulaeformis carr. In west of Shanxi province. Bulletin of Soil and Water Conservation**,** **33**(2)**,**151-155.
140. Wang Min, Su Yong, Yang Rong, et al. 2013. Allocation patterns of above- and belowground biomass in desert grassland in the middle reaches of Heihe River, Gansu Province, China. Chinese Journal of Plant Ecology**,** **37**(3)**,**209-219.
141. Hong Tao, Wu Chengzhen, Lin Yongming, et al. 2012. Biomass characteristics in the Arbor Layer of Aleurites Montana plantation. Journal of Mountain Science**,** **30**(6)**,**648-654.

142. Li Zhoukun. 2012. Species selection and biomass allocation pattern of 4-year-old acacia species on Fujian southeast coastal sandy land. Jour of Fujian Forestry Science and Technology, **39**(3),15-18.
143. Hu Yongyan. 2012. Effect of different slope positions on the growth and biomass distribution of Phoebe bournei plantation aged twenty-one. Journal of Jiangsu Forestry Science & Technology, **39**(4),6-8.
144. Wang Tianbo. 2012. A study on individual tree biomass models of Pinus Massoniana plantation of Jiangle Fujian. Beijing Forestry University.
145. Zhang Jinghui, Qi Yu, Wang Yan, et al. 2012. The effects of grazing on individual-level and community-level biomass allocation in the stipa baicalensis steppe. Journal of Beijing Normal University, **48**(3),270-275.
146. Ming Angang, Jia Hongyan, Tao Yi, et al. 2012. Biomass and its allocation in a 28-year-old Mytilaria laosensis plantation in southwest Guangxi. Chinese Journal of Ecology, **31**(5),1050-1056.
147. Gao Chengjie. 2012. Study on biomass allocation and nutrient cycling characteristics of Azadirachta indica and acacia auriculiformis plantations in dry-hot valley. Chinese Academy of Forestry.
148. Lei Lei. 2012. Distribution character of alpine shrubs biomass along an elevation gradient in Qilian mountains. Gansu Agricultural University.
149. Qin Lin, He Yongjun, Li Zhiyong, et al. 2012. Allocation pattern of biomass and productivity for three plantations of Castanopsis hystrix, Pinus massoniana and their mixture in south subtropical area of Guangxi, China. Scientia Silvae Sinicae, **47**(12),17-21.
150. Liu Guangying, Zhao Guohua, Wang Guangmei, et al. 2011. Allocation pattern of Larix principis rupprechtii biomass. Heibei Journal of Forestry and Orchard Research, **26**(3),222-226.
151. Liu Yanhui, Wang Yanhui, Yu Pengtao, et.al. 2011. Biomass and its allocation of the main vegetation types in Liupan Mountains. Forest Research, **24**(4),443-452.
152. Cai Qinglou. 2011. Study on biomass and distribution rate of Cinnamomum glanduliferum（Wall）Nees in different slopes. Hubei Academy of forestry, (3),15-18.
153. Chen Qingxia. 2011. Biomass distribution pattern of 8-year-old Acacia fasciculata plantation at different slope positions. Forestry Survey and Design, (1),107-110.
154. Huang Zhihong, Tian Dalun, Kang Wenxing, et al. 2011. Dynamic of biomass distribution in first rotation of Chinese fir plantation in Huitong county, Hunan province. Journal of Central South University of Forestry & Technology, **31**(5),37-43.
155. Wang Xiuyun. 2011. Distribution of carbon storage for Larix olgensis plantation of different stand ages. Beijing Forestry University.
156. Cao Jixing. 2011. Above- and belowground carbon pools in different ages of Chinese Pine and Oriental arborvitae plantation forests in northern mountain areas of Beijing. Beijing Forestry University.

157. Huang Qinzhong. 2010. Research on the biomass distribution of Magnolia officinalis plantation under different slope. Jiangxi Forestry Science and Technology**,** (6)**,**16-18.

158. Zhang Shiguan, Liu Jian, Huang Kaiyong, et al. 2010. Biomass and distribution patterns of Pinus massoniana plantation in Northwest Guangxi. Guangxi Forestry Science**, 39**(4)**,**189-192.

159. Chen Shijun, Wei Meiyu. 2011. Biomass allocation and estimation model of stem tuber of Oncidium grandiflorum. Journal of Qiannan Normal University for Nationalities**, 30**(3)**,**47-49.

160. Ma Weiling, Shi Peili, Li Wenhua, et al. 2010. The change of individual plant traits and biomass allocation in alpine meadow with elevation variation on the Qinghai-Tibetan Plateau. Science China Life Sciences**, 40**(6)**,**533-543.

161. Xu Zhengzhou. 2010. Study on spatial distribution pattern of the biomass and plant diversity in Larix princicpis-rupprechtii Mayr. plantation in Yanshan Mountainous regions. Agricultural University of Heibei.

162. Liu Bin. 2010. The study on the individual biomass and biomass allocation pattern of Piuns tarbulaeformis of Mt. Helan, China. Northwest A & F University.

163. Wen Shizhi. 2010. Study on the density, storage and distribution of carbon in Alnus cremastogyne artifical forest system. Central South University of Forestry & Technology.

164. Mo Lifeng, Tang Jianwei, Dao Xiangsheng, et al. 2010. Influence of stand densities on the biomass and seed yield of Jatropha curcas plantations. Journal of Central South University of Forestry & Technology**, 30**(4)**,**61-68.

165. Li Xiaona. 2010. Biomass allometry and allocation of common understory in a natural secondary forest in Maoershan, Northeast China. Northeast Forestry University.

166. Pan Yumei, Tang Saichun, Cen Yanxi, et al. 2010. Biomass allocation on the modules of aster subulatus Michx. population at flowering stage. Journal of Tropical and Subtropical Botany**, 18**(2)**,**176-181.

167. Ding Zhuang. 2010. Study on the carbon allocation and storage of Pinus koraiensis plantation. Northeast Forestry University.

168. Li Yanqiong, Zhang Shaowei, Li Depeng, et al. 2009. A study of the relation of Sophora davidiana biomass and soil moisture content in the arid valley of the upper Minjian river. Journal of Sichuan Forestry Science and Technology**, 39**(4)**,**17-22.

169. Zhu Hongru. 2009. Study on efficiency of mixed forest with Fokienia hodginsii and michelia macelurei in productivity and soil nutrient. Fujian Agriculture & Forestry University.

170. Hong Tao. 2009. Study on carbon storage of Aleurites montana plantation ecosystem. Fujian Agriculture & Forestry University.

171. Chen Xiaoyue. 2009. Stand Biomass and its distribution of mixed forests of Alnus cremastogyne and Cunninghamia lanceolata. Jiangxi forestry science and technology**,** (1)**,**14-17.

172.	Liu Guizhou, Cai Chuangtao, Luo Yuan, et al. 2009. Biomass allocation and biomass estimation models of Rauvolfia vomitoria and Rauvolfia verticillate (Apocynaceae). Acta Botanica Yunnanica, **31**(1),63-66.
173.	Peng Longfu. 2008. Preliminary study on the biomass of Phoebe bournei plantation with different density and site condition. Journal of Fujian Forestry Science and Technology, **35**(4),15-18.
174.	Gao Yongheng, Chen Huai, Luopeng, et al. 2008. Effect of razing intensity on biomass of Alpine meadow and its allocation in the northwestern Sichuan. Journal of Ecology and Rural Environment, (3),26-32.
175.	Qin Wuming, He Bin, Qin shiying. 2008. Biomass and productivity in Acacia crassicarpa plantation. Journal of Northwest Forestry University, **23**(2),17-20.
176.	Qi Jianfei, Tang Jianwei. 2007. Biomass and its allocation pattern of monsoon rain forest over limestone in Xishuangbanna of southwest China. Chinese Journal of Ecology, **27**(2),167-177.
177.	Jiang Zeihui, Fang Shaohui, Feng Huixiang, et al. 2007. Biomass and Distribution Patterns of Populus × xiaohei Plantation in Sandy Land of North China. Scientia Silvae Sinicae, **43**(11),15-20.
178.	Lv Xiaotao, Tang Jianwei, He Youcai, et al. 2007. Biomass and its allocation in tropical seasonal rain forest in Xishuangbanna, southwest China. Journal of Plant Ecology, **31**(1),11-22.
179.	Deng Kunmei, ShiPeili, YangZhenlin, et al. 2006. Biomass allocation and net primary productivities at treeline ecotone on the Changbai mountains, northeast China. Journal of Natural resources, (6),942-948.
180.	Fan Houbao, Li Yanyan, Su Bingqiang, et al. 2006. Allocation pattern of biomass and productivity in the mixed uneven-aged stands of Masson' s pine and hardwood species. Acta Ecologica Sinica, (8),2463-2473.
181.	Lin Fang. 2006. Biomass characteristics and allocation of Castanopis fargesii natural stand in Wuyishan mountains. Acta Agriculturae Universitatis Jiangxiensis, **28**(1),74-77.
182.	Wu Zemin, Wu Wenyou, Lu Bing, et al. 2003. Biomass and nutrient accumulation and distribution of Pinus taiw anensis in Dabie Mountains of Anhui Province. Journal of Anhui Agricultural University, **30**(3),294-298.
183.	Fang Hua, Kong Hua. 2003. Study on biomass and its allocation of different density Loblolly Pine. Journal of Fujian College of Forestry, **23**(2),182-185.
184.	Zan Qijie, Wang Yongjun, Liao Baowen, et al. 2001. Biomass and net productivity of Sonneratia apetala, S. caseolaris Mangrove man-made forest. Journal of Wuhan Botanical Research, **19**(5),391-396.
185.	Ying Jinhua, He Zongming, Fan Shaohui, et al. 2001. Research on biomass and its distribution of a 29-year-old, first rotation Chinese first plantation. Journal of Fujian college of Forestry, **21**(4),339-342.
186.	Long Xuewu, Ye Gongfu, Xu Junsen, et al. 1999. Study on density effect of Casuarina equisetifolia plantation biomass. Jour of Fujian Forestry Science and Technology, **26**(1),21-23.

187. Luo Shuifa. 1990. Study on the biomass of 3-year-old Eucalyptus Caphylla Plantatio. Journal of Fujian College of Forestry, **19**(3),279-281.
188. Zhou Guangyi, Zeng Qingbo, Lin Mingxian, 1997. Biomass and nutrient allocation in Manglietia hainanensis plantation ecosystem at Jianfengling. Forestry Research, **10**(5),4-8.
189. Ai Xunru, Zhou Guanglong. 1996. Study on the biomass of Chinese Fir plantation's ecosystem in the North edge of median sub-tropic. Hubei Forestry Science and Technology, (2),17-20.
190. Zheng Yanming. 1996. Biomass and its allocation in a 28-year-old Castanopsis kawakamii plantation. Journal of Fujian College of Forestry, **16**(2),114-118.
191. Huang Xihe. 1988. Study on biomass, nutrient element content and distribution of Cunninghamia lanceolata plantation in Xijiang area. Forestry Survey and planning in Central South China, (1),13-21.
192. Li Xiangdong. 1984. Preliminary study on biomass and nutrient element distribution of Phoebe plantations in different sites. Journal of Guangxi Agricultural University, (2),88-100.
193. Luo Hang. 2021. Charaeteristics of nutrient biological cycle in Melaleuca alternifolia plantations with different stand ages. Central South University of Forestry and Technology.
194. Huang Kangtian. 2019. The research on growth regularity and biomass on distribution characteristics of 30-year-old Keteleeria cyclolepis flous. Central South University of Forestry and technology.
195. Chen Qingzhong. 2019. Study on growth and biomass of Cunninghamia lanceolata and Liquidambar formosana mixed stand. Forestry Survey and design, **39**(3),55-58.
196. Wang Ning, Fu Fengzhen, Cheng Haotian, et al. 2019. Estimation of forest biomass and carbon stock in the Urban Forest Park of Daqing city. Journal of Heilongjiang Bayi Agricultural University, **31**(3),9-14.
197. He Mingzeng. 2019. Research the growth characteristics and ecological stoichiometry of Caesalpinia sappanplantation. Guangxi University.
198. Liu Yuhui. 2019. Biomass and under canopy plant diversity of a mixed forest of Chinese fir (Cunninghamia lanceolata) and Cyclobalanopsis gracilis. Subtropical Agriculture Research, **14**(2),104-109.
199. Zhao Zhang. 2017. Biomass and carbon storage of Cunninghamia lanceolata and Castanopsis hystrix plantation in south subtropical China. Guangxi University.
200. Zhang Chenyueer. 2017. Study on the biomass and carbon storage of Pinus massinnina plantation in Qingyang lake national forest park. Central South University of Foredtry & Technology.
201. Yang Junsong. 2016. Study on biological productivity, nutrient characteristics and water conservation function of Betula luminifera plantation in Northwest Guangxi. Guangxi University.
202. Deng Ping. 2015. Characters of biomass and carbon density in vegetation of

Pinus massoniana plantation. Jiangxi Agricultural University.
203. Chen Danfen, Liu Wanqiu, Huang Guoxian, et al. 2014. Study on biomass and energy utilization of late pine. Academic Field, (9),21-24.
204. Shen Jinliang, Feng Yonglin, Zhang Jianshe, et al. 2014. Research on the biomass of four major tree species in the vegetation restoration pattern area of Beichuan Nature Reserve. Journal of Sichuan Forestry Science and Technology, **35**(4),27-31.
205. Liu Yanwen, Wang Xiyang, Ma Lvyu, et al. 2003. Biomass productivity of Larix principis-rupprechtii plantation of different age classes. Heibei Journal of Forestry and Orchard research, **28**(4),339-343.
206. Zhang Xuewu. 2012. Study on ecosystem services of planted P.tabulaeformis forest in the loess hilly region. Northwest A&F University.
207. Shi Shandan, Zhao Pengwu, Zhou Mei, et al. 2012. Biomass and carbon storage of the secondary forest (Populus davidiana) at different stand growing stages in southern Daxinganling temperature zone. Ecology and Environmental Sciences, **21**(3),428-433.
208. Weng Xian. 2011. Study on the biomass distribution regular patterns of Cunninghamia lanceolata and Manglietia yuyuanensis mixed forest. Anhui Forestry Science and Technology, **37**(3),18-21.
209. Huang Qinzhong. 2011. Biomass distribution of 9-year-old Taiwania flousiana plantation in different slope. Journal of Anhui Agriculture Science, **39**(4),2113-2125.
210. Zhao Yong, Fan Wei, Wu Mingzuo, et al. 2009. The nutrient allocation and cycling pattern in the Platycladus orientalis plantation in Hilly Region of Taihang Mountains. Journal of Soil and Water Conservation, **23**(2),143-147.
211. He Bin, Dao Hailin, Huang Hengchuang, et al. 2008. Changing regularity of biomass and productivity of Tauwania fiousiana plantation. Journal of Northeast Forestry University, **36**(9),17-18.
212. Liang Ni, Wang Weibin, Tian Kun, et al. 2006. Biomass distribution characteristics of 4 and 13 years old Betulaalnoides plantations. Journal of West China Forestry Science, **35**(4),118-122.
213. Zhang Zhijun, Wang Yanhui, Yuan Yuxin, et al. 2006. Study on the biomass structure and distribution of natural secondary forest of Pinus massoniana. Journal of Agricultural University of Hebei, **29**(5),37-43.
214. Xie Tianshi. 2006. The productivity and nutrient cycling of Metasequoia glyptostroboides plantation ecosystem in the mountain area of south. Fujian Agriculture and Forestry University.
215. Chen Jianhua. 2006. Study on biomass of cypress Fraxinus. Fujian Agricultural Science and Technology, (4),61-63.
216. Liu Chunhua. 2006. Comparison of biomass productivity between Sloanea sinensis plantation and Cunninghamia lanceolata piantation. Sobtropical Agriculture Research, **2**(1),29-32.
217. Huang Dianzhong. 2006. Biomass characteristics of secondary forestry community of Cyclobalanopsis chungii in the lower Mingjiang River.

Protection Forest Science and Technology, (1),16-18.
218. Feng Lizhi, Zheng Zheng, Tang Jianwei, et al. 2005. Biomass of tropical secondary Mallotus paniculatus forest in Xishuangbanna. Chinese Journal of Ecology, **24**(3),238-242.
219. Yao Yingjiu. 2003. Biomass and nutrient accumulation and distribution of camphor plantation. Central South Forestry College.
220. Yao Yingjiu, Kang Wenxing, Tian Dalun, et al. 2003. Study of the biomass and productivity of Cinnamomum camphora plantation. Journal of Central South of Restry University, **23**(1),1-5.
221. Zhao Kun, Tian Dalun. 2000. Study of the biomass and productivity of Nature Chinese Fir Stand in Huitong County. Journal of Central South Forestry University, **20**(1),7-13.
222. Qiu Guiyun. 1999. Study on the individual tree biomass of 20-year-old Cunninghamia lanceolata plantation under different site conditions in North Fujian. Journal of Fujian Forestry Science and Technology, **26**(3),21-23.
223. Wu Shoude, Ye Gongfu, Pan Huizhong, et al. 1996. Structural characteristics of biomass of Casuarina equisetifolia plantation. Shelter forest technology, (1),21-24.
224. Chen Deyang. 2022. Study on biomass of 36 years old natural secondary forest of Castanopsis hystrix. Horticulture & Seed, **42**(1),44-47.
225. Xu Rui, Xie Jiang, Yan Xiangshuai, 2021. Comparison on biomass and carbon storage of Rubber plantation with other types of forest in Xishuangbanna. Chinese Journal of Tropical Crops, **42**(4),1145-1153.
226. Zhang Lingkai, Wang Yan, Zhao Yanxia, et al. 2021. Ecosystem carbon storage and within-system distribution in Castanea mollissima plantations at different ages on the southern slope of Yan Mountain. Journal of Hebei Agricultural University, **44**(2),79-85.
227. Song Liangyou, He Gongxiu. 2021. Distribution characteristics of biomass and carbon storage of Cunninghamia lanceolata plantation in different age stages. Hunan Forestry Science and Technology, **48**(1),71-75.
228. Hui Liudi, Liu Fansheng, Mo Shaozhung, et al. 2021. Biomass accumulation and productivity changes of Cunninghamia lanceolata plantations in Northwest Guangxi. Subtropical Agriculture Research, **17**(2),78-83.
229. Zhang Rishi, Tang Leihou, Cao Jizhao, et al. 2021. Biomass Productivity and Distribution of Pinus massoniana Plantations with Different Stand Ages in Southwestern Guangxi. Guangxi Forestry Science, **50**(4),373-377.
230. Yu Hang, Pan Jianghong, Yang Liusheng, et al. 2021. Relationship between biomass of dominant herbaceous plants and soil physical properties in a dry-hot valley area of the Jinsha River. Chinese Journal of Applied and Environmental Biology, **27**(4),884-892.
231. Zhang Rishi, Huang Zhenge, He Bin, et al. 2021. Biomass accumulation and productivity changes of Taiwania flousiana plantation at different age stages in northwestern Guangxi, southern China. Journal of Beijing Forestry

University, **43**(11),20-27.
232. Wang Jixing, Xu Picong, Li Manqing, et al. 2019. Biomass of Cunninghamia lsnceolata piamtation in Tengchong city of Yunnan province. Protection Forest Science and Technology, (12),22-23.
233. Tang Zhiming, Liu Bingxiang, Qu Yu, et al. 2020. Study on biomass and carbon storage of arbor layers in typical soil and water conservation forests in Taihang mountain range in Hebei Province. Forest Resources Management, (1),102-107.
234. Tian Jiase, Ma Shiming, Wang Zhibo, et al. 2020. Study on Biomass and Carbon Storage of Larix principis rupprechtii in Sumushan of Inner Mongolia. Journal of Inner Mongolia Forestry Science and Technology, **46**(1),21-24.
235. Wu Hongyang, Liang Naipeng. 2020. Study on Biomass of Castanopsis kawakamii Hayata Plantation. Anhui Agricultural Science Bulletin, **26**(19),70-72.
236. Liao Guoli, Duan Jie, Jia Zhongkui, et al. 2020. Distribution characteristics of carbon storage in Larixolgensis plantation ecosystem of different ages in Eastern Liaoning Province. Journal of Northeast Forestry University, **48**(11),8-13.
237. Liu Qiu, Wu Jiyou, Yang Shouzhi, et al. 2018. Analytical wood analysis and biomass investigation of Cyclobalanopsis glauca plantation. Journal of Central South University of Forestry & Technology, **38**(12),22-29.
238. Zheng Zhizheng. 2018. Effects of different planting patterns of Fokienia hodginsii and Schima superba on biomass in coastal mountainous areas of Southern Fujian. Forestry Survey and design, **38**(4),21-23.
239. Xue Chunquan, Xu Qihu, Lin Liping, et al. 2019. Biomass growth models for individual tree of main indigenous broadleaf tree species in Guangdong Province. Journal of South China Agricultural University, **40**(2),65-75.
240. Zhang Ligang, Lu Guiping. 2019. Study on the biomass of chinese Fir plantation in state owned Gao Feng Forest Farm of Guangxi. Forestry science and technology information, **51**(1),43-45.
241. Zhang Hongmei. 2019. Determination of biomass of 23-year-old rice row plantation. Journal of Green Science and Technology, (12),30-31.
242. Wang Xinying, Shi Junhui, Liu Maoxiu. 2018. Nutrient accumulation and biomass characteristics of different-aged natural forests of Populus euphratica in the Tarim river basin. Journal of northwest Forestry University, **33**(3),45-51.
243. Zhou Xiaolin. 2018. Study on the biomass of 15-year-old Masson Pine in Guangxi Huangmian Forest Farm. South China Agriculture, **12**(14),96-98.
244. Wei Xu, Qin Dewen. 2016. Studies on the growth，biomass and productive forces of Toonailiate plantation. Journal of Fujian Forestry Science and Technology, **43**(4),38-42.
245. Chen Dongshen, Sun Xiaomei, Zhang Gongshou, et al. 2016. Biomass, carbon storage and nutrient characteristics in Larix kaempferi plantation at

different stand ages. Chinese Journal of Applied Ecology, **27**(12),3759-3768.
246. Zhang Ying, Wu Pengfei, Zou Xianhua, et al. 2016. A comparative study on growth and biomass production of different broadleaved tree species plantations in Northern Fujian Province. Subtropical Agriculture Research, **12**(4),231-235.
247. Li Jiabo, Wei Qiusi, Wu Qingbiao, et al. 2017. Biomass and carbon storage of Keteleeria davidiana natural forest in south subtropics mountain. Huibei Forestry Science and Technology, **46**(1),14-19.
248. Xu Yongxing. 2017. Net Productivity of Cunninghamia lanceolata and Michelia maudiae in Mixed and Pure Stand. Journal of Zhejiang for Science and Technology, 37(1),47-50.
249. Huang Shajia, Li Teihua, Wen Shizhi, et al. 2017. Study on growth rule and biomass distribution pattern of Cyclobalanopsis glauca. Journal of Central South University of Forestry & Technology, **37**(3),57-62.
250. Liu Zhizhou, Ning Chen, Yan Wende, et al. 2017. Three kinds of pine forest biomass and carbon storage research in karst landform. Journal of Central South University of Forestry & Technology, **37**(10),105-111.
251. Wang Bin, Wei Tianxing, Liu Zhao, et al. 2017. Characteristics of nutrient cycling and ecosystem structure of Larix principis-rupprechtii in hilly loess plateau. Research of Soil and Water Conservation, **24**(6),45-51.
252. Wang Liyan, Liu Guangzheng, Lin Xiaofan, et al. 2015. Biomass of natural secondary evergreen broad-leaved forest in Dexing area of Jiangxi Province. Journal of West China Forestry Science, **44**(6),115-121.
253. Fang Xinrong. 2015. A study of accumulation of biomasses and nutrition elements in mixed forests of Chinese fir (Cunninghamia lanceolata) and Schima superba. East China Forest Management, **29**(4),39-42.
254. Song Yali, Han Hairong, Kang Fengfeng. 2016. Study on biomass and carbon storage with age-chronosequence in Pinus tabulaeformis forest in Taiyue mountain of Shanxi province. Research of Soil and Water Conservation, **23**(1),29-33.
255. Huang Qunzhen, Zhang Caize. 2016. Biomass of 44-year-old Manglietia glauca plantation in Gaofeng forest farm of Guangxi Zhuang autonomous region. Protection forest Science and Technology, (2),4-6.
256. Fu Mengyao, He Junzhao, Song Lianghong, et al. 2016. The dynamic tree layer biomass of forest vegetation in the mountainous areas of Sanmenxia. Henan Science, **34**(3),332-337.
257. Leng Tianfeng, Lou Li, Pan Zhen, et al. 2016. A preliminary report on the biomass of seed production stand of Styrax suberifolius. Guizhou Forestry Science and Technology, **44**(1),17-20.
258. Zheng Wei, Shen Wenhui, Tan Yibo, et al. 2016. Biomass and its distribution pattern of Castanopsis hystrix plantation in southeastern Guangxi. Journal of Central South University of Forestry & Technology, **36**(5),85-87.
259. Gan Guichun, Wang Wei, Wang Xiaoning, et al. 2016. Biomass and carbon storage of Eucalyptus Artificial Stand. Forest Inventory and Planning,

41(02),34-37+42.

260. Duan Pingguo, Yang Chuan, Li Chaoguo, et al. 2016. Study on the guangxi state run Yachang forest farm of Betula luminifera plantation biomass. Forestry Science and Technology Information, **48**(2),55-57.

261. Li Jianming. 2016. Biomass and carbon storage patterns of two natural secondary forests in Maoer Mountain. Inner Mongolia Forestry Investigation and Design, **39**(3),27-29.

262. Yang Weixing, He Bin, Lu Kaicheng, et al. 2016. Biomass and productivity of Eucalyptus urophylla E. grandis plantations in Continuous Age Sequences in Southwest Guangxi. Agriculture research and application, (3),6-11.

263. Zhao Jing, Kang Fengfeng, Han Hairong, et al. 2016. Carbon sequestration capacity of a 31-year-old Larix principis-rupprechtii plantation on Mt. Taiyue, Shanxi Journal of Arid Land Resources and Environment, **30**(10),110-115.

264. Lu Lixia. 2016. Study on biomass and stand structure characteristics of five Acacia species along the coast of eastern Fujian. Forestry Survey and design, **36**(2),22-26.

265. Luo Yuesheng. 2016. The growth and biomass of Eucalyptus dunnii plantation. Fujian Forestry, (3),36-39.

266. Huang Jinling. 2016. The biomass and productivity of T. Flousiana plantation on south mountain. Journal of Fujian Forestry Science and Technology, **43**(3),161-164.

267. Bi Huitao, Wang Bingyan, Yang Hongzhen, et al. 2014. Study on the dynamic change of the tree layer biomass of the community Quercus aliena var. acutiserrata-Pinus armandii of Baiyunshan mountain in Henan province. Journal of Henan Agricultural University, **48**(6),736-740.

268. Wu Jinghua, Tian Feng, Gui Lingjian, et al. 2014. A preliminary study on biomass of Eucalyptus urophylla× E. grandis plantation in Nanning Qipo Forest farm. Journal of Guangxi Academy of sciences, **30**(4),233-237.

269. Huang Bichang, He Bin, Zhou Yanping, et al. 2015. The growth regularity and biomass productivity of Betula alnoides plantation in Northwest Guangxi. Forestry science and technology development, **59**(1),123-126.

270. Xu Shancai. 2014. Biomass structure characteristics of natural secondary forest of Pinus massoniana in Wuyishan area. Journal of Wuyi University, **33**(5),10-13.

271. Tang Xiaocheng, Zhang Jianping, Lu Shiguang, et al. 2015. Preliminary study on biomass of 14-year-old sightseeing wood plantation. Journal of Green Science and Technology, (6),9-10.

272. Luo Fenrong. 2015. Studying on vertical distribution of individual tree biomass of Fokienia hodginsii (Dunn). Fujian agricultural science and technology, (5),23-26.

273. Gao Nan. 2015. Research on the biomass of cunninghamia konishii Hayata and Chinese fir plantations. Journal of Fujian Forestry Science and

Technology, **42**(2),46-49.
274. Bai Chunlei, Nie Zhenzhen, Jia Dewen. 2015. Study on biomass and productivity of 12-year-old brandy. Journal of Green Science and Technology, (8),36-37.
275. Wan Liqin. 2015. Biomass structure characteristics of Keteleeria fortunei plantation from Yongchun, Fujian province. Subtropical Plant Science, **44**(3),228-230.
276. Lin Xiaofang. 2014. Research of vertical distribution characteristics of Castanopsis fissaplantations'biomass. Jiangxi Forestry Science and Technology, **42**(1),10-11.
277. Yan Li, Liu Xiaolu, Qin Wuming, et al. 2014. Biomass of Pinus yunnanensis var. tenuifolia natural forest in Baise Region of Guangxi Province. Journal of West China Foretry Science, **43**(3),134-138.
278. Shao Mingxiao, Wen Shizhi, He Congxiu, et al. 2014. The biomass structure characteristics of P. bournei (Hemsl.) Yang plantation in different ages. Journal of Central South University of Forestry & Technology, **34**(6),44-48.
279. Yang Yujiao, Chen Yunming, Cao Yang. 2014. Carbon density and distribution of Pinus tabulaeformis plantation ecosystem in Hilly Loess Plateau. Acta Ecologica Sinica, **34**(8),2128-2136.
280. Xuan Huochao, Qin Dewen, Yang Qinchao, et al. 2014. Biomass and productivity of Quercus acutissima plantation. Guizhou Agricultural Science, **42**(6),120-123.
281. Wang Nihong, Cao Meng, Li Dan. 2014. Tree layer biomass distribution and carbon storage capacity of Larix olgensis plantation. Bulletin of Botanical Research, **34**(4),554-560.
282. Zhou Qunying, Chen Shaoxiong, Han Feiyang, et al. 2013. Studies on biomass and energy characteristics of short rotation energy plantations of Eucalyptus urophylla E. grandis. Journal of Tropical and Subtropical Botany, **21**(1),45-51.
283. Xuan Zhilong, Zhang Qichang, Ge Lili, et al. 2013. Biomass structure and distribution of Korean Larch plantations. Forest Resources Management, (1),53-57.
284. Yang Xujing. 2013. Determination and analysis of biomass of Magnolia officinalis artificial medicinal raw material forest. Forestry Survey and design, (1),82-84.
285. Yang Yanfu. 2013. Growth, biomass and soil nutrient characteristics of Pinus massoniana broad-leaved mixed forest. Forestry Survey and design, (1),77-81.
286. Li Xiao, Yu Zhiyong, Xu Zhongqi, et al. 2013. Study on biomass of different age Larix principis-rupprechtii plantations. Heibei Journal of Forestry and Orchard Research, **28**(3),226-232.
287. Yang Yang, Ran Fei, Wang Genxu, et al. 2013. Biomass model and carbon storage of Pinus yunnanensis on Tibet Plateau of China. Chinese Journal of Ecology, **32**(7),1674-1682.

288. Ma Wei, Sun Yujun. 2013. Biomass under different densities for middle-aged Larix olgensis plantation. Journal of Northeast University, **41**(8),13-17.
289. Wu Min, Li Songhai, Qin Wuming, et al. 2013. Study on biomass and productivity of 11-year-old Michelia hedyosperma plantation. Journal of West China Forestry Science, **42**(4),46-51.
290. Sun Baogang, Chenfei, Wang Jianmin, et al. 2012. Biomass distribution pattern of different parts of Pinus Yunnanensis with different diameter class. Forest Research, **25**(1),71-76.
291. Chen Kaiyun, Hu Yangang, Song Riqin, et al. 2012. Study on biomass distribution of Betula luminifera at different ages. Forest By-Product and Speciality in China, (2),17-20.
292. Gao Yanping, Pan Mingliang, Ding Fangjun, et al. 2012. Study on biomass and net productivity of natural secondary forests of Betula luminifera in west Guizhou. Journal of Central South University of Forestry & Technology, **32**(4),55-60.
293. Hu Shasha, Zhang Yutao, Li Jianmei, et al. 2012. Characteristics of spatial distribution of the biomass of populus alba var pyramidalis bge. Xinjiang Agricultural Sciences, **49**(6),1059-1065.
294. Zhu Shaomu. 2012. Study on biomass structure of Chinese fir deep mountain Michelia mixed stand. Anhui Agriculture Science Bulletin, **18**(13),121-123.
295. Cheng Xiaoqin, Han Hairong, Kang Fengfeng. 2012. Biomass, carbon accumulation and its partitioning of a Pinus tabulaeformis plantation ecosystem in Shanxi Province, China. Chinese Journal of Ecology, **31**(10),2455-2460.
296. Fang Jiangping. 2012. Study on biomass and productivity of Picea likiangensis var. linzhiensis forest in Nanyigou of Tibet. Forest Research, **25**(5),582-589.
297. Qin Wuming, Qiu Bingfa, Tang Jing, et al. 2011. Study on the biomass and growth law of Paramichelia baillonii plantation. Journal of Fujian College of Forestry, **31**(2),110-114.
298. Cai Qinglou. 2011. Research on the biomass of Pinus massoniana plantations. Journal of Anhui Agriculture Science, **39**(12),7122-7124.
299. Li Junzhen, Qin Wuming, Qin Yu, et al. 2011. Study on the biomass and productivity of Manglietia giauce. Journal of Fujian Forestry Science and Technology, **38**(1),1-5.
300. Xu Wen, Hu Haibo, Zhou Changhai, et al. 2011. Biomass structure and distribution characters of Pinus massoniana in eastern reigon of Anhui province in China. Journal of Central South University of Forestry & Technology, **31**(6),111-115.
301. Huo Changfu, You Wenzhong, Zhang Huidong, et al. 2011. Biomass and net primary productivity of Quecus mongolica plantation in Binglashan Mountains in Liaoning Province. Journal of Liaoning Forestry Science and Technology, (4),4-6.

302. Ming Angang, Tao Yi, Wu Guangzhi, et al. 2011. The study of the growth and biomass of MytiLaria laosensis plantation in different slope positions. Chinese Agricultural Science Bulletin**, 27**(28)**,**90-93.
303. Jing Wenmao, Liu Xiande, Zhao Weijun, et al. 2011. Study on biomass and net productivity of typical forest stand in the Qilian Mountains. Journal of Gansu Agricultural University**, 46**(6)**,**81-85.
304. Fan Jiyou, Tan Dewen, Jiang Lin, et al. 2011. Research on the tree biomass and productivity of Cryptomeria fortunei Hooibrenk plantation. Journal of Fujian Forestry Science and Technology**, 38**(4)**,**1-5.
305. You Wenzhong, Huo Changfu, Xing Zhaokai, et al. 2011. Biomass and net primary productivity of Larix olgensis plantation in Bingla Mountains, Northeast China. Journal of Shenyang Agricultural University**, 42**(5)**,**565-569.
306. Xue Bo, Yan Deren, Hu Xiaolong, et al. 2010. Biomass characteristics of Poplar plantation in sandy land. Journal of Inner Mongolia Forestry Science and Technology**, 36**(1)**,**11-14.
307. Liang Youxiang, Qin Wuming, Wei Zhongmian, et al. 2010. The growth regularity, biomass and productivity of Acacia melanoxylon in the southwest of Guangxi. Jour of Forestry Science and Technology**, 37**(2)**,**1-4.
308. Lin Wenfeng, Lin Dixian. 2010. Study on biomass and productivity characteristics of Masson Pine Plantation. Journal of Heilongjiang Vocational Institude of Ecological Engineering**, 23**(5)**,**19-21.
309. Huang Yong, Jia Jing, Li Songchan, et al. 2010. Research on growth law, biomass and productivity of Acaxia cincinnata plantation. Journal of Anhui Agriculture Science**, 38**(22)**,**12050-12053.
310. Liang Youxiang, Wei Zhongmian, Yu Guicheng, et al. 2010. A study on biomass and productivity of michelia macclurei dandy plantation in southeast of Guangxi. Forestry Science and Technology Development**, 24**(5)**,**45-49.
311. Han Feiyang, Zhou Qunying, Chen Shaoxiong, et al. 2010. Study on biomass and energy of two different aged Eucalyptus stands. Forest Research**, 23**(5)**,**690-696.
312. Yan Jiafeng, Guan Qingwei, Deng Songqiu, et al. 2009. Study on biomass and productivity of platycladus orientalis plantation in Yunlong Mountain of Xuzhou. Forestry Science and Technology Development**, 23**(2)**,**48-50.
313. Huang Lijuan. 2009. Study on biomass and carbon density of Acacia mangium plantation. Guangxi Tropical Agriculture**,** (2)**,**8-12.
314. Zhang Guishan. 2009. Study on growth and biomass of Chinese fir mixed forest. Anhui Agriculture Science Bulletin**, 15**(21)**,**131-132.
315. Deng Qiuxiang, Zhao Ying, Wu Lujun, et al. 2008. Biomass productivity of a 12-year-old Cunninghamia Lanceolata plantation in Daqingshan of Guangxi. Guangxi Forestry Science**, 37**(4)**,**187-190.
316. Ma Mingdong, Jiang Hong, Liu Yuejian, et al. 2008. Biomass, carbon content, carbon storage and their vertical distribution of Phoebe bourmei artificial

stand. Scientia Silvae Sinicae, (3),34-39.
317. Li Qingyun, Wan Meng, Fan Wei, et al. 2008. Study on biomass and productivity of Populus plantation in the Huanghuaihai Plain. Heinan Science, (4),434-437.
318. Yi Liping, Wen Shizhi, Wang Zhenzhen, et al. 2008. Biomass and productivity of Liquidambar formosana hance. Journal of Central South University of Forestry & Technology, (2),50-53.
319. Fang Jiangping, Xiang Wenhua. 2008. Biomass and its distribution of a primeval Abies georgei var.smithii Forest in Sejila Mountain in Tibet Plateau. Scientia Silvae Sinicae, **44**(5),17-23.
320. Xie Weidong, Wen Yuanguang, Liang Hongwen, et al. 2008. Biomass and productivity of Casuarina equisetifolia shelter belt on coastal sandy land in Guangxi. Protection Forest Science and Technology, (5),6-8.
321. Huang Changchun. 2008. Biomass and productivity of a 26-year-old hybrid yellow-poplar plantation in Shaxian, Fujian. Journal of Fujian Forestry Science and Technology, **35**(3),10-13.
322. Wang Xiangyu, Hu Dong, He Jingheng, et al. 2007. Biomass research of Fagus engleriana and Quercus aliena var. acuteserrata forest in Shennongjia Foest District. Journal of Capital Normal University, **28**(2),62-67.
323. Cai Yong. 2007. A comparison of the biomass and their distribution patterns between the mixed forest of Chinese fir and michelia macclurei and pure Chinese fir plantation. Journal of Fujian Forestry Science and Technology, **34**(2),48-52.
324. Guo Zijuan, Song Xide, Zhao Honggang, et al. 2007. Biomass and soil characteristics of Hippophae rhamnoides and Platycladus orientalis mixed forest and its root system distribution. Bulletin of Soil and Water Conservation, **27**(3),18-23.
325. Li Shaoqing. 2007. Study on spatial distribution pattern of biomass in 22-year-old Pinus massoniana forest. Fujian Agricultural Science and Technology, (4),76-77.
326. Xie Weidong, Wen Yuanguang, Zhou Minyi, et al. 2007. Biomass and productivity of Eucalyptus urophylla plantation in new cultivated areas. Journal of Central South University of Forestry & Technology, **27**(5),13-18.
327. Chen Meigao. 2006. Studies of biomass characteristics of Pinus massoniana plantation on different ages. Journal of Fujian College of Forestry, **26**(4),332-335.
328. Zhang Renhao. 2006. Biomass and productivity of 14-year-old Zenia insignis plantation. Journal of Fujian Normal University, **22**(4),102-106.
329. Luo Daqing, Zheng Weilie, Wang Jiangsheng, et al. 2004. Biomass and yield of Betula platyphylla population in Mila Mount of Tibet. Chinese Journal of Applied Ecology, **15**(4),1329-1333.
330. Liu Aiqin, Liu Chunhua, Ma Xiaoqing, et al. 2004. Study on biomass production of Cyclobalanopsis chungii plantation. Journal of Fujian College of Forestry, **24**(4),294-297.

331. Chen Dexiang, Li Yide, Luo Tishou, et al. 2004. Study on biomass and net primary productivity of Podocarpus imbricatus plantation in Jianfengling, Hainan Island. Forest Research**,** (5)**,**598-604.

332. Wu Xilin. 2003. Study on the biomass and structure of mixed forests of Acasia crassicarpa and Casuarina equsetif olia. Journal of Fujian College of Forestry**, 23**(3)**,**236-239.

333. Zhu Wanze, Xue Jianhui, Wang Jinxi, et al. 2002. Studies on the stand biomass and the distribution of nutrients of Alnus formosana plantation. Journal of Nanjing Forestry University**, 26**(2)**,**15-20.

334. Lu Shengping. 2002. Research on the Taiwania flousiana gaussen and the biomass and law of growth of planted forest in Xingdoushan Nature Reserve. Journal of Hubei Institute for Nationalities**, 20**(4)**,**14-17.

335. Huang Yueqiong, Wu Xiaofeng, Han Weidong, et al. 2002. A study on biomass of Sonneratia apetala Artificial Stands. Acta Agriculturae Universitatis Jiangxiensis**, 24**(4)**,**533-536.

336. Liu Yucui. Wu Mingzuo, Guo Zongming, et al. 2001. Studies on biomass and net production of Quercus acutidentata forest in Baotianman Nature Reserve. Acta Ecologica Sinica**, 21**(9)**,**1450-1456.

337. Guo Jianfeng. 2001. Study on biomass and soil fertility of mixed stands of Pinus massoniana and Michelia macclurei in mountain area of southern Fujian. Protection Forest Science and Technology**,** (3)**,**4-6.

338. Jiang Zongkai. 2000. Biomass and its allocation in a 34-year-old Forkienia hodginsii plantation. Journal of Northeast Forestry University**, 28**(6)**,**16-19.

339. Yang Zongwu, Tan Fanglin Xiao Xiangxi, et al. 2000. A study on biomass of Fokienia hodginsii plantation. Scientia Silvae Sinicae**,** 36(1)**,**120-124.

340. Ren Hai, Peng ShaoLin, Xiang YanCi, et al. 2000. Biomass and net primary productivity in an acacia mancium plantatin in Heshan,GuangDong,China. Acta Phytoecologica Sinica**,** (01)**,**18-21.

341. Huang Tao, Zhong Qiuping, Peng Xiaoyan, et al. 2009. Study on biomass and productive forces of artificial Liriodendron Chinese. Jiangxi Forestry Science and Technology**,** (05)**,**4-9.

342. Li Xuehua, Li Xiaolan, Jiang Deming, et al. 2009. A comparative study of the individual biomass and Modular Biomass of 70 herbaceous species found in the Horqin sandy Land. Arid Zone Research**, 26**(02)**,**200-205.
